# Adoption of 3D-Printed Food in Romania: Price Perception as a Key Determinant of Consumer Acceptance

**DOI:** 10.3390/foods14244306

**Published:** 2025-12-14

**Authors:** Iuliana Petronela Gârdan, Mihai Ioan Roșca, Daniel Adrian Gârdan, Mihai Andronie, Laura Daniela Roșca, Carmen Adina Paștiu

**Affiliations:** 1 Faculty of Psychology and Education Sciences, Spiru Haret University, 060821 Bucharest, Romania; 2 Faculty of Marketing, Bucharest University of Economic Studies, 010374 Bucharest, Romania; mirosca@ase.ro; 3 Faculty of Economic Sciences, Spiru Haret University, 060821 Bucharest, Romania; se_gardand@spiruharet.ro (D.A.G.); mihai_a380@spiruharet.ro (M.A.); 4 Doctoral School of Marketing, “Dunărea de Jos” University of Galați, 800008 Galați, Romania; 5 Doctoral School of Entrepreneurship, Engineering and Business Management, National University of Science and Technology Politehnica Bucharest, 060042 Bucharest, Romania; 6 Management—Marketing Faculty, Romanian-American University, 012101 Bucharest, Romania; laura.rosca@rau.ro; 7 Faculty of Economic Sciences, 1 Decembrie 1918 University, 510009 Alba-Iulia, Romania

**Keywords:** 3D food printing, consumer acceptance, price perception, technology adoption, sustainable food innovation, personalized nutrition, structural equation modeling

## Abstract

Three-dimensional printed food has rapidly positioned itself at the intersection of food technology and personalized nutrition, opening up new perspectives for sustainable production, creative customization, and more efficient resource use. Although global interest in this innovation continues to grow, consumer acceptance remains largely underexplored in Central and Eastern Europe. This study analyzes how Romanian consumers approach the adoption of 3D-printed food by applying an extended UTAUT2 framework to a sample of 608 urban respondents. Using structural equation modeling, it examines the influence of expected effort, performance expectancy, social influence, and perceived compatibility on adoption intention, while price perception is introduced as a key mediating variable—a novel and meaningful contribution to the literature on food technology acceptance. Given the non-probabilistic sampling design, the difficulties encountered in measuring Hedonic Motivation and Facilitating Conditions, and the early diffusion stage of 3D food printing in Romania, the present work should be viewed as a robust exploratory investigation based on Structural Equation Modeling (SEM) among urban Romanian consumers, providing first empirical evidence on 3D-printed food acceptance in Eastern Europe rather than definitive conclusions for the entire population. The results highlight that utilitarian and social factors are decisive: expected effort enhances perceived performance, while performance, social influence, and compatibility significantly strengthen perceptions of price fairness. In turn, price perception strongly predicts consumers’ behavioral intention to adopt 3D-printed food. Hedonic motivation and facilitating conditions were not statistically significant and were therefore removed from the final model. These findings show that, in emerging food markets, consumers tend to make adoption decisions based more on rational value assessments than on novelty or convenience. The study contributes to theory by embedding price perception into the UTAUT2 framework and to practice by identifying the key elements that can boost market readiness—transparent pricing and closer alignment with consumer values. By filling an important gap in the empirical literature from Eastern Europe and focusing on price as a cognitive bridge between technological and psychological drivers, this paper offers a timely and relevant contribution to ongoing research on consumer perception and acceptance of food innovations. For Eastern European food innovation research, this study provides one of the first quantitative analyses of 3D-printed food acceptance that explicitly links technology-related beliefs to price perception in a regional, price-sensitive context.

## 1. Introduction

The rapid advancement of additive manufacturing technologies has opened new frontiers in various industries, including the food sector, where 3D printing has emerged as a disruptive innovation with significant potential to transform production, distribution, and consumption patterns. Beyond its technological novelty, 3D food printing addresses pressing global challenges such as food personalization, sustainability, and efficient resource utilization, making it an area of growing interest for both researchers and practitioners. These developments occur in a broader socio-economic context marked by shifting dietary preferences, the need for sustainable solutions, and the increasing demand for customized nutritional options [1,2]. In this respect, 3D food printing can be regarded not only as a technological achievement but also as a pathway toward sustainable and consumer-oriented food systems, where innovation meets nutritional personalization and resource efficiency.

The technology that allows the 3D printing process to come to life dates back to the 1970s, when Wyn Swainson patented a process capable of producing 3D objects using two laser beams [1,2]. Subsequent technological advancements enabled successful applications across various domains—ranging from the first Stereolithography-based commercial printers in 1988, to micro-level casting in the 1990s, and the open-source RepRap project in 2005. In terms of organic materials, one of the first major applications appeared in 2014, when NASA experimented with 3D-printed food for astronauts, signaling the transition of this technology from polymers to edible matter. This trajectory underscores how 3D printing, initially conceived for industrial purposes, has evolved into a versatile tool for food innovation, with applications spanning from high-end gastronomy to medical nutrition.

In this context, 3D food production refers to the layer-by-layer deposition of nutritional materials in predetermined sizes, quantities, and shapes, using computer-aided design [2].

While more experimental developments such as 4D and 5D food printing further extend these possibilities by allowing printed foods to change shape or be produced with more complex geometries [3,4,5], such formats are still far from everyday consumption. Accordingly, the present study focuses on 3D-printed foods that are most likely to reach commercial and food-service applications in the near term and investigates how consumers evaluate their usefulness and value-for-money in this context.

The scope of 3D food printing also includes the use of unconventional ingredients—such as algae, fungi spores, insects, buckwheat, or lupin seeds—as sustainable protein sources. These components not only support environmental goals but also align with emerging consumer preferences for health-focused, ethical, and high-protein diets. Mate-rials used for 3D food printing can be classified as native printable materials, traditional non-printable food ingredients, and novel alternatives. Recipes range from conventional preparations to experimental combinations, making this technology a unique platform for culinary innovation and value chain reconfiguration [6]. From a consumer behavior perspective, this material and technological diversity intersects with core issues of acceptability, as unfamiliar formats and ingredients can challenge norms of taste, safety, and perceived value.

Consumer acceptance of 3D-printed food is a complex, multi-dimensional process, shaped by a wide array of economic, cultural, psychological, and technological factors. Compared to conventional food systems, 3D-printed food offers mass customization, greater consumer control, and the possibility to tailor meals to individual health needs and preferences with precise caloric and nutritional profiles. It enables creative combinations of textures, shapes, and flavors that respond to contemporary dietary expectations. Furthermore, in special contexts—such as space missions, remote work environments, or emergency settings—3D-printed food offers practical, scalable, and cost-effective solutions.

This process of adoption fits within broader technology acceptance frameworks. In Central and Eastern Europe, where disposable incomes and food price sensitivity remain critical, these dynamics are particularly salient for understanding how consumers evaluate novel food technologies. Starting from the UTAUT2 model (Unified Theory of Acceptance and Use of Technology, version 2), this study proposes and tests seven hypotheses that identify key predictors of behavioral intention to adopt 3D-printed food, emphasizing the mediating role of price perception. While international research has employed UTAUT and TAM (Technology Acceptance Model) in this domain [7,8], there is a conspicuous lack of empirical studies focusing on Romania. A comprehensive review of major academic databases (Web of Science, Scopus) revealed no prior empirical research examining consumer attitudes toward 3D-printed food in this country. The only related findings on digitalization are found in the agricultural field [9], which indirectly highlights the novelty of this topic and reinforces the need to explore how Romanian consumers perceive innovative food technologies. This gap is particularly important because Eastern European food markets often differ in terms of digital adoption, price sensitivity, and openness to novelty, compared to Western markets. Moreover, Romania’s case allows for a unique exploration of how consumers balance functional value and experiential novelty in assessing food innovations—especially when price perception mediates acceptance.

Building on this gap, the present study integrates price perception as a central mediating variable in an extended UTAUT2 framework. This approach provides a nuanced understanding of how functional expectations, social influence, and compatibility translate into behavioral intention—an aspect largely overlooked in existing research on food technology adoption.

The main objective of this study is to analyze the key determinants of consumers’ behavioral intention to adopt 3D-printed food, with particular emphasis on the mediating role of price perception—a construct rarely tested in prior research. Specifically, the model examines how expected effort, expected performance, social influence, facilitating conditions, hedonic motivation, and perceived compatibility affect adoption intention. The study offers two main contributions: (1) it is the first empirical examination of consumer acceptance of 3D-printed food in Romania, and (2) it sheds light on the economic and social mechanisms—particularly those related to price perception—that influence adoption decisions. Given the reliance on a convenience, urban and technology-connected sample, the present research is explicitly positioned as an exploratory investigation that offers first SEM-based evidence on 3D-printed food acceptance among Romanian consumers, opening the way for more representative follow-up studies.

This work also aligns with ongoing scientific efforts to explore the consumer ac-acceptance and market potential of 3D-printed foods, especially in digitally driven and sustainability-conscious food systems.

The remainder of the paper is structured as follows: Section 2 presents the literature review and the proposed theoretical model. Section 3 describes the methodology, including sampling and construct measurement. Section 4 reports the empirical findings and discusses theoretical and practical implications. Section 5 concludes with the study’s contributions, limitations, and future research directions.

## 2. Theoretical Framework and Hypothesis Development

The acceptance and adoption of 3D-printed food is a complex process that can be analyzed within the general framework of technology adoption models such as the Technology Acceptance Model (TAM) and the Unified Theory of Acceptance and Use of Technology (UTAUT/UTAUT2). These models emphasize the role of performance expectations, effort, social influence, and other factors in shaping users’ behavioral intentions toward new technologies.

In the case of food innovations, consumer acceptance is shaped not only by perceptions of utility and ease of use, as predicted by classical adoption models, but also by cultural, symbolic, and hedonic dimensions. Research on novel foods such as plant-based proteins, edible insects, and cultured meat has shown that adoption decisions often depend on trust, social legitimacy, and perceptions of naturalness rather than purely on technological performance. For example, Mancini and Antonioli (2022) [10] highlight how Italian consumers balance curiosity and skepticism when evaluating insect-based and cultured meat products, while De Koning et al. (2020) [11] demonstrate that both drivers and inhibitors of acceptance are rooted in cultural and hedonic perceptions as much as in utilitarian evaluation. As such, constructs derived from technology adoption theory require contextual adaptation when applied to food technologies, especially those involving novel formats or ingredients.

In order to successfully analyze the specifics of the adoption of 3D food preparation technology, it is useful to briefly review how technology acceptance has been researched in the specialized literature. From a chronological perspective, and considering the increasingly complex conceptualization of technology acceptance, it is natural to start with the work of Ajzen and Fishbein, who in 1977 developed the Theory of Reasoned Action (TRA). The two researchers concluded that a person’s behavior is mainly determined by the intention to behave in a certain way [12].

Intention, in turn, is influenced by both the individual’s attitude toward the behavior (positive or negative) and the social norms to which the individual relates. These norms reflect the perceived value placed on the behavior by important others within the individual’s social circle. According to the TRA, attitudes are shaped by beliefs about the outcomes of adopting a behavior, while subjective norms are based on motivation to comply with others’ expectations and on normative beliefs. In essence, the TRA frames individual behavior as a rational decision-making process, in which the implications of actions are systematically considered before acting.

Building on the TRA, Ajzen (1991) developed the Theory of Planned Behavior (TPB), which incorporates behavioral, normative, and control beliefs to explain behavior [13]. From the TPB, the Technology Acceptance Model (TAM) was derived, aiming to clarify technology adoption behavior and becoming one of the most widely used models in technology acceptance research.

Beyond the TAM, several other models have been proposed to explain technology adoption in different contexts [14,15,16,17,18,19]. However, given the objectives of this study and the need to keep the conceptual framework closely aligned with the variables tested empirically, we focus on TAM- and UTAUT2-based perspectives, which explicitly integrate performance and effort expectations, social influence and price-related evaluations as key drivers of behavioral intention. A significant evolution in technology acceptance theory came with the development of the Unified Theory of Acceptance and Use of Technology (UTAUT) and its extension, UTAUT2, proposed by Venkatesh et al. (2012) [20]. By integrating earlier models—TRA, TAM, Motivational Model (MM), TPB, combined TAM and TPB (C-TAM-TPB), Model of PC Utilization (MPCU), IDT, and SCT—the UTAUT offers a more comprehensive and predictive framework than TAM, as also noted in recent empirical applications [21].

While the UTAUT2 has been successfully applied across various technology domains, its adaptation to the food innovation sector remains relatively scarce. Moreover, most existing models do not explicitly address how consumers evaluate price fairness when adopting novel food technologies.

This study addresses that gap by introducing price perception as a mediating variable between functional and social predictors and behavioral intention. This integration offers a refined lens to understand how consumers process both utility and cost when forming adoption decisions—particularly in culturally distinct and economically sensitive markets such as Romania.

Building on the theoretical foundations outlined above, the present study adapts the variables proposed in the UTAUT2 model to the specific context of 3D-printed food adoption. In doing so, the model departs from standard UTAUT2 applications by incorporating price perception as an explicit mediating construct between selected antecedents (expected performance, social influence, compatibility) and behavioral intention. This addition reflects the assumption that in the case of food innovations—especially those requiring cognitive or sensory adjustment—consumers may not directly translate perceived benefits into adoption, but rather filter them through an evaluation of price fairness and value. By situating price perception at the center of the decision-making process, the model better captures the economic and psychological pathways relevant to consumer acceptance in emerging food systems.

The following section details each construct and formulates the corresponding research hypotheses.

We will begin by addressing to a basic variable for the intended model—expected effort. Expected effort has been consistently associated in the scientific literature with the degree of ease implied by the use of a technology. In essence, the ease with which a system can be operated assumes a minimal effort from the user. A technology is perceived as useful if the consumer can operate it without difficulty, or if it’s functioning is free of effort [22]. Venkatesh also defines ease of use as the degree of simplicity associated with the effective operation of a system [20]. This perceived ease is linked by consumers to positive emotions and satisfaction in usage, with each user who perceives an application as clear and simple tending to use it more frequently [23].

Within the TAM2 model, the discussion continues, as in TAM1, to address perceived ease of use, which is directly related to both the intention to use and perceived usefulness [24,25]. The objective usefulness of a new system for users depends largely on the level of efficiency they develop as they interact with it [26].

The literature consistently supports the idea that the expected effort required to use a system is closely connected to its perceived utility for the individual—in other words, to the performance the user expects from the system and its ability to help them accomplish tasks more effectively. In the context of 3D-printed food, consumers who perceive this technology as easy to use are more likely to expect it to meet their needs related to personalized nutrition, food customization, or innovative culinary experiences [27,28].

Based on other research summarized in the literature, the relationship between low effort expectations and high-performance expectations is consistently supported in the field of emerging food technologies. When users perceive the interaction with a technology as intuitive and free of operational barriers, their confidence in the system’s ability to deliver the expected results increases. This effect has been observed both in the case of mobile applications for personalized nutrition and in the use of smart cooking equipment, where the easy-to-understand design and simplified process of use lead to a better assessment of anticipated performance [29]. In addition, studies indicate that the perception of reduced effort reduces technological anxiety, facilitating a more positive evaluation of perceived usefulness and, implicitly, of expected performance [30]. This relationship becomes even more important in the context of 3D-printed food, where consumer familiarity with the technology is still low, and the initial experience, if free of difficulties, can directly influence how the technology’s potential to meet demands for personalized nutrition, culinary creativity, or sustainability is perceived. As a final remark, in a meta-analysis on the UTAUT2 model, Tamilmani et al. (2021) found that in most of the studies analyzed, expected effort significantly influenced expected performance, particularly in contexts involving the development or application of a new technology [31].

Considering these arguments, the first research hypothesis can be formulated as follows:

**H1.**  *Expected Effort (EE) has a positive influence on Expected Performance (EP) regarding the use of 3D-printed food.*

The second variable taken into consideration refers to expected performance. Venkatesh et al. defines Expected Performance (EP) as the degree to which a system is perceived to improve user productivity or deliver tangible performance gains [20]. In the case of 3D-printed food, this concept reflects the perceived utility, functionality, and added value that consumers anticipate when considering this type of innovation, whether this refers to personalization of meals, precise nutritional optimization, improved convenience, or contributions to sustainability.

When consumers perceive strong performance benefits, they tend to evaluate price more favorably, as higher expected utility often offsets perceptions of high cost. This is consistent with the idea that perceived value results from a trade-off between the benefits and the sacrifices associated with acquiring a product [32]. For example, Hassoun highlights that perceived performance benefits strongly influence consumer acceptance of novel food technologies, particularly when these benefits are aligned with tangible needs such as minimizing waste or enhancing dietary personalization [33]. Similarly, Feng et al. (2022) [28] show that the usefulness of 3D food printing in terms of customization and time-saving significantly influences consumer evaluations, leading to greater willingness to pay.

Halassi, Semeijn, and Kiratli (2019) add that functional benefits such as ingredient control, product consistency, and creative flexibility increase the perceived worth of the technology, making the price appear more justified [34]. These findings align with the perspective of the UTAUT2 framework, which states that greater performance expectancy strengthens the perceived overall value of a product or service [31].

Price fairness has been consistently identified as a critical determinant in consumer evaluations of food innovations. While hedonic or novelty-based drivers may generate initial curiosity, consumers often rely on utilitarian and economic assessments to form longer-term adoption intentions. Wang et al. (2024) [35] confirm that innovation-adoption characteristics, including trust and perceived value, strongly shape purchase intentions for alternative proteins, indicating that economic justification often outweighs hedonic appeal in the decision-making process Similarly, Zeithaml (1988) [36] conceptualized price as both a sacrifice and a signal of value, while Varela and Fiszman (2013) [37] emphasized that perceptions of price fairness significantly condition willingness to adopt novel food products. Therefore, integrating price perception into UTAUT2 allows a more accurate understanding of consumer decision-making in the case of 3D-printed food.

Considering these arguments, it is reasonable to state the following hypothesis:

**H2.**  *Expected Performance (EP) has a positive influence on Price Perception (PP) regarding 3D-printed food.*

The acceptance of an innovation often depends on what people who matter to the consumer say or do. The opinions and reviews of friends, family members, colleagues, or influencers can tip the balance in favor of or against the adoption of a technology, including in the food industry [38]. In the digital environment, these influences are quickly transmitted through online reviews, social media posts, and personal recommendations, creating a social validation effect that can shape price perceptions [39].

In the case of 3D-printed foods, endorsements from credible sources or groups with which the consumer identifies can build trust in the quality and benefits of the product. If these messages emphasize features such as personalization, sustainability, or innovation, they can create a more favorable perception of the price, even when it is above the market average. In essence, a consumer may be willing to pay more if the product has a positive image maintained by people or communities they respect.

Research shows that this social influence can work in several ways. It can be through conformity—when someone adopts a behavior to align with the expectations of a group; internalization—when the beliefs of a significant referent become part of one’s own belief system; or identification—when using the product contributes to the individual’s status and image in their group [40,41]. In all these cases, the link between social influence and price perception is mediated by the credibility of the source and the social relevance of the product.

From a marketing perspective, social influence identification—the situation where using or purchasing a product aligns the consumer with a valued group or trend—can have a particular impact on 3D-printed foods. If early adopters and admired individuals publicly flaunt their consumption of these products, price perceptions can shift to an “aspirational premium” level, which can sustain demand even at higher prices [34].

Thus, in the context of 3D-printed food, positive recommendations from key individuals and membership in communities open to innovation can transform an initially perceived high price into one perceived as justified.

Taking into consideration all of the above, we may advance the third hypothesis:

**H3.**  *Social Influence (SI) positively influences Price Perception (PP) toward 3D-printed food.*

Facilitating conditions reflect the extent to which consumers perceive that they have the necessary resources, infrastructure, and knowledge to use a new technology [42]. In the context of food technologies, these conditions may include access to reliable internet connections, user-friendly devices, clear instructions, and technical support. When such facilitating conditions are available, consumers are more confident in using the technology and are more likely to perceive the price of the product as fair and justified. Conversely, when facilitating conditions are weak or absent, even an objectively reasonable price can be seen as too high, because the consumer anticipates difficulties in adoption.

For 3D-printed food, facilitating conditions are particularly important because the technology is still in an early adoption phase. Studies emphasize that clear tutorials, demonstrations, and accessible support systems enhance consumer trust and reduce uncertainty, which in turn allows a higher acceptance of premium pricing [43]. Moreover, when the surrounding ecosystem provides adequate logistical and technical infrastructure—such as the availability of cartridges, maintenance services, and integration with existing kitchen devices—consumers interpret the price not just as a cost but as a justified investment in convenience and innovation [44].

Recent analyses also highlight that facilitating conditions can transform potential barriers into value-enhancing elements. For instance, the availability of reliable supply chains and transparent communication from producers reduces perceived risks and strengthens the association between price and quality [45]. These findings suggest that facilitating conditions not only enable adoption but also actively shape the way consumers evaluate and interpret pricing.

Therefore, it is theoretically consistent and empirically supported to assume that favorable facilitating conditions positively influence consumers’ price perception of 3D-printed food, by lowering uncertainty, increasing trust, and enhancing perceived value. Thus, H4 can be advanced:

**H4.**  *Facilitating Conditions (FC) positively influence Price Perception (PP) toward 3D-printed food.*

Hedonic motivation is defined as the fun or pleasure derived from using a technology, and it has been shown to play an important role in determining technology acceptance and use [46]. In internet services related research, hedonic motivation—often conceptualized as perceived enjoyment—has been found to directly influence technology acceptance and use [47,48].

In the context of 3D-printed food, hedonic motivation is particularly relevant, since part of the attraction of this innovation lies not only in efficiency but in the novelty, creativity, and playful aspects of the consumption experience. For consumers, experimenting with personalized shapes, textures, or even colors of printed food can generate enjoyment and curiosity, which in turn can enhance willingness to accept higher prices for such unique experiences. Research confirms that early adopters are especially driven by hedonic factors, although this effect tends to diminish over time as the focus shifts toward efficiency and performance [31].

This dynamic is important for 3D-printed food because the playfulness and pleasure of testing innovative recipes or designs can justify a premium price point, particularly in the early adoption phase. For instance, Palau-Saumell et al. (2019) found that hedonic motivation directly and positively affects intentions to use mobile applications for restaurant reservations, showing that consumers value not only utility but also the enjoyment derived from interaction [21]. By analogy, the interactive and creative potential of 3D-printed food can reinforce the perception that its higher price is justified by the hedonic experience offered.

Furthermore, demographic factors may moderate the impact of hedonic motivation. Studies based on UTAUT2 indicate that younger consumers, particularly men with lower experience, are more sensitive to hedonic benefits, while older and more experienced users rely less on enjoyment and more on habit or functionality [20]. In the case of 3D-printed food, this implies that hedonic motivation could play a stronger role in shaping price perception among younger, trend-sensitive consumers, who may associate innovation with pleasure and status.

Thus, hedonic motivation (HM) not only stimulates curiosity and trial but also frames the higher price of 3D-printed food as an acceptable “premium for enjoyment,” reinforcing our hypothesis.

**H5.**  *Hedonic Motivation (HM) positively influences Price Perception (PP) toward 3D-printed food.*

Perceived compatibility (matching with the individual’s values, eating habits and consumption routine) matters for price through the mechanism of perceived value: when 3D-printed food “fits” with the diet, needs and social context (e.g., nutritional personalization, sustainability, adapted textures), people see a greater benefit and are more willing to consider the price fair or even accept a premium. Empirically, perceived compatibility has been shown to increase both the intention to consume and the willingness to pay a premium for 3D-printed food—a result that directly suggests a more favorable price perception when the product aligns with the consumer’s expectations and lifestyle [27].

Compatibility can also be cultivated through how we deliver information: messages that link 3D-printed food to relevant benefits (e.g., personalization, ingredient control, health) improve attitudes and reduce cognitive friction, which, in terms of price, mitigates the feeling of “sacrifice” and shifts the balance towards “value” [49].

In addition, acceptance is strongly contextual: when the consumption situation “fits” (place, company, occasion), openness to innovative foods—including 3D-printed ones—increases; in such compatible contexts, higher prices can be interpreted as justified by the perceived benefit [50].

The literature review on 3D-printed foods also shows that fit with functional needs (e.g., medical diets, textures for dysphagia) enhances perceived value, a direct antecedent of more favorable price evaluations [51].

Therefore, we formulate the following hypothesis:

**H6.**  *Perceived Compatibility (PC) positively influences Price Perception (PP) toward 3D-printed food.*

Within the UTAUT2 framework, price perception reflects the cognitive balance consumers make between expected benefits and the cost they bear; when perceived benefits outweigh costs, adoption intentions rise [20]. Research on digital applications also shows that not only the monetary dimension matters but also a price-saving orientation: consumers’ value being able to compare prices and obtain savings, which strengthens their intention to use the technology [21].

For 3D-printed food, recent findings indicate that perceived product value and lifestyle compatibility drive both consumption intentions and willingness to pay a premium price, which directly links favorable price perception to adoption [27]. The classical price literature further emphasizes the dual role of price—as a financial sacrifice and as a signal of quality—helping explain why positive value inferences can translate into stronger behavioral intentions [32,52].

Evidence from food-service technologies points the same way: when consumers perceive clear savings or a favorable value-to-cost ratio, intention to use increases [53]. Conversely, when benefits are unclear or costs appear high, the effect of price perception on intention diminishes, underscoring the need to tie pricing to tangible benefits [54]. In the case of 3D-printed food, clear labeling and consumer information help reinforce fairness perceptions and justify price levels [28]. Recent work also highlights that branding and labeling practices play a decisive role in shaping consumer trust and market acceptance, especially when it comes to novel or less familiar food products [55].

Accordingly, we propose the hypothesis.

**H7.**  *Price Perception (PP) has a positive influence on Behavioral Intention (BI) to adopt 3D-printed food.*

The hypotheses advanced above—H1–H7—allow us to build a conceptual (theoretical) model that highlights the cumulative effect of the factors taken into account (Expected Performance (EP), Social Influence (SI), Facilitating Conditions (FC), Hedonic Motivation (HM), Perceived Compatibility (PC) and Expected Effort (EE)—mediated by Expected Performance (EP) on Price Perception (PP). The model integrates variables from UTAUT 2 and TAM 2, having as its characteristic the fact that it places price perception as a connecting element between the listed factors and the behavioral intention (BI) of consumers to adopt this new technology. In our empirical specification, this connecting role is implemented as a mediation pattern: expected performance, social influence, facilitating conditions, hedonic motivation, perceived compatibility and expected effort are specified as antecedents of price perception, and price perception in turn predicts behavioral intention, so that these factors influence intention primarily through their impact on price perception.

**Figure 1:**
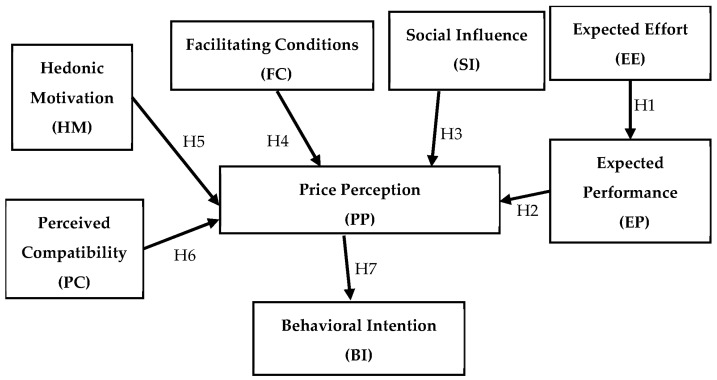
Proposed conceptual model.

This integrative approach emphasizes the crucial role of price perception as a determinant of consumers’ willingness to adopt 3D-printed food. The proposed theoretical model is illustrated in Figure 1.

Overall, by extending UTAUT2 with constructs particularly salient in the food domain—such as price perception and compatibility—this framework provides a novel perspective on the adoption of 3D-printed food. This integration allows us to capture both the technological and the socio-economic dimensions of acceptance, thereby enriching technology adoption theory while offering practical insights into consumer behavior toward emerging food innovations. Chia et al. (2024) [56], further illustrate that barriers such as perceived “unnaturalness” can significantly influence consumer evaluations of novel foods, reinforcing the need to adapt technology adoption models to food-specific contexts. A distinctive feature of our framework is that price perception is not only added as another direct predictor of intention, but is explicitly modeled as a mediating construct between UTAUT2-type antecedents and behavioral intention—an arrangement that, to our knowledge, has not yet been empirically tested for 3D-printed foods in Eastern European markets.

In building this conceptual model, we deliberately focused on those UTAUT2 constructs that are most proximal to the decision to try an innovative food rather than to continue using an already adopted technology. Expected effort and expected performance capture the core cognitive evaluation of how demanding and how beneficial 3D-printed food is perceived to be, with EE specified as an antecedent of EP in line with Venkatesh et al. (2012) [20]. Social influence reflects injunctive and descriptive norms around food innovations, which are particularly salient in cultures where family and peer evaluations shape dietary experimentation. Facilitating conditions and hedonic motivation were initially included because they summarize, respectively, perceived availability of resources/infrastructure and anticipated enjoyment, both of which have shown effects in prior UTAUT2 applications in food and hospitality settings. Perceived compatibility was added as a food-specific construct to capture the fit of 3D-printed food with consumers’ habits, values and dietary routines, extending evidence from Moore and Benbasat (1991) [57] and Yang et al. (2024) [27]. Finally, price perception was positioned as a mediating mechanism between these antecedents and behavioral intention, based on the assumption that in a price-sensitive, emerging market consumers may translate perceived performance, social endorsement and lifestyle fit into adoption only if the resulting offer is evaluated as fair and good value for money [52]. Other UTAUT2 constructs, such as habit, were not retained in the present model because our cross-sectional design and the very early diffusion stage of 3D-printed food in Romania do not allow for a meaningful operationalization of repeated use or established usage patterns.

Beyond generic technology acceptance factors, the literature on food decision-making emphasizes that adoption is a multi-stage process in which emotional responses, perceived naturalness and safety considerations play a central role. For example, Abdullah et al. (2024) show that food choices shape emotional experience and purchase intentions in the case of a traditional product such as nasi lemak, highlighting how taste expectations, symbolic meanings and affective reactions jointly drive the decision process [58]. In the case of innovative foods, constructs such as food neophobia, perceived (un)naturalness and safety or health concerns have been repeatedly identified as critical predictors of acceptance. These variables can either reinforce or undermine the influence of utilitarian drivers, and their omission from adoption models risks underestimating the psychological complexity of food-related decisions.

In line with this stream of research, the present study deliberately focuses on technological and socio-economic determinants (effort, performance, social influence, compatibility and price perception), but future extensions should explicitly integrate food-specific psychological variables such as neophobia, perceived naturalness and risk perceptions, in order to capture the full adoption process.

## 3. Materials and Methods

### 3.1. Sample and Data Collection

The empirical analysis was based on a quantitative survey conducted among Romanian consumers in order to investigate the antecedents of behavioral intention to adopt 3D-printed food. Romania was chosen as a case study because empirical research on consumer acceptance of 3D-printed food in Central and Eastern Europe is virtually absent. As an emerging market with increasing exposure to sustainable and personalized food solutions, Romania provides a valuable context to examine how cultural and economic specificities influence adoption intentions.

The questionnaire was designed following the guidelines of previous research in technology acceptance and food innovation [20,59]. The items were adapted to the specific context of 3D-printed food and measured on a seven-point Likert scale, ranging from 1 (“total disagreement”) to 7 (“total agreement”).

Data were collected using an online structured questionnaire distributed through specialized survey platforms and social media channels between April and May 2024. A non-probabilistic convenience sampling strategy was applied, which is considered appropriate for exploratory studies in emerging research areas [60]. After data screening and cleaning procedures, a total of 608 valid responses (n = 608) were retained for further analysis, with a structure that can be seen within Table 1. However, the use of online channels and convenience sampling implies that the sample over-represents younger, urban and digitally connected respondents. As a result, the findings cannot be generalized to the entire Romanian population, particularly older adults and residents of rural areas, but should instead be interpreted as reflecting the attitudes of urban consumers with higher exposure to technology and sustainable food discourses.

The demographic profile of the respondents shows a balanced gender distribution (49.3% male and 50.7% female), while the majority were residents of urban areas (80.3%). Regarding age distribution, 33.4% of respondents were between 18 and 25 years, followed by 28.3% in the 26–35 years category, 13.7% between 36 and 45 years, 15.3% between 46 and 55 years, and 9.4% over 56 years. In terms of education, most respondents reported having completed secondary studies (41.9%), followed by undergraduate studies (29.3%) and postgraduate studies (19.2%). Income levels were also well distributed, with 43.1% earning between 3501 and 7000 RON per month, reflecting the middle-class purchasing power in Romania.

The survey also included behavioral aspects, such as respondents’ health interest and frequency of fast-food consumption, to ensure a better contextualization of their perceptions regarding innovative food technologies. Notably, more than half of the respondents declared being “very interested” in their health (55.3%) and a majority (55.9%) reported frequently attending fast-food outlets, indicating an openness toward both health-oriented and convenience-driven food behaviors.

Participation in the survey was voluntary and anonymous, and informed consent was obtained from all respondents prior to data collection. The study complied with ethical research standards. To ensure measurement validity, internal consistency was evaluated using Cronbach’s alpha, while convergent and discriminant validity were assessed through AVE values and the Fornell–Larcker criterion.

Given the novelty of 3D-printed food, this sample provides relevant insights into the attitudes of younger, digitally literate, and urban-oriented consumer groups, which represent the most likely early adopters of disruptive food technologies [27,49].

### 3.2. Measurement of Constructs

The questionnaire was originally designed in Romanian, ensuring linguistic and cultural appropriateness for the target population. The measurement of the constructs used in this research was based on well-established scales adapted from prior studies in technology acceptance and consumer behavior, to fit the specific context of 3D food printing. At the beginning of the questionnaire, respondents were presented with a brief written description of 3D food printing, summarizing the basic principles of the technology and typical examples of printed foods, in order to ensure a minimum level of conceptual understanding. No images, videos or other visual stimuli were provided, so all responses are based on this textual explanation and on respondents’ self-reported familiarity with the concept.

All items were measured on a seven-point Likert scale, ranging from 1—“total disagreement” to 7—“total agreement”. A pilot test with 30 respondents was conducted to assess item clarity, wording and preliminary reliability (Cronbach’s α > 0.80 for all multi-item scales). Based on participants’ comments, minor adjustments were made to simplify technical expressions related to 3D-printed food and to ensure that price-related items were clearly framed in terms of perceived fairness rather than absolute cost. The pilot sample was not included in the final analysis.

Below we will briefly present the details for every construct from the model, while items used for measurement within the level of applied questionnaire being explained within the Table 2:

Effort Expectancy (EE). Five items capturing perceived ease of learning and using the technology, adapted from Venkatesh & Davis (2000) [26] and Frank & George (2023) [62].

Performance Expectancy (EP). Four items reflecting the extent to which using 3D-printed food is perceived to improve outcomes (efficiency, convenience, nutritional benefits), adapted from Venkatesh et al. (2012) [20].

Social Influence (SI). Three items assessing the perceived influence of important others (family, peers, opinion leaders) on using 3D-printed food, adapted from (Venkatesh & Davis, 2000) [26].

Facilitating Conditions (FC). Four items tapping the availability of resources and support (information, access, device readiness) to use the technology, adapted from Venkatesh et al. (2012) [20].

Hedonic Motivation (HM). Four items capturing enjoyment/pleasure from interacting with 3D-printed food, adapted from Palau-Saumell et al. (2019) [21].

Perceived Compatibility (PC). Three items assessing the fit with personal values, habits, and lifestyle/dietary routines, adapted from Moore & Benbasat (1991) [57] and Yang et al. (2024) [27].

Price Perception (PP). Four items reflecting perceived price fairness/value and price-saving orientation related to 3D-printed food, adapted from Palau-Saumell et al. (2019) [21] and Yang et al. (2024) [27].

Behavioral Intention (BI). Five items capturing willingness and intention to adopt 3D-printed food, adapted from Venkatesh & Davis (2000) [26] and Venkatesh et al. (2012) [20].

### 3.3. Data Analysis Procedure

Data were analyzed using IBM SPSS Statistics 28 and AMOS 28 for Structural Equation Modeling (SEM). Reliability and validity were assessed through Cronbach’s alpha, Composite Reliability (CR), Average Variance Extracted (AVE), and the Fornell–Larcker criterion.

Data analysis was conducted in two stages. In the first stage, the measurement model was evaluated to verify reliability and validity criteria. In the second stage, the structural model was tested using SEM to assess the hypothesized relationships (H1–H7). Model fit was evaluated based on indices such as CFI, TLI, RMSEA, and χ^2^/df, following the recommendations of Hair et al. (2019) [63]. Multicollinearity was checked through VIF values (<3.0), confirming the absence of redundancy among constructs.

## 4. Results

### 4.1. Overview of the Research Model

Before proceeding with the model testing, an overview of the research context and conceptual design is provided to ensure clarity on how the proposed hypotheses (H1–H7) were operationalized. The model presented in this article is part of a larger research project that set out to explore how consumers think and behave when faced with three very different trends in the food sector: eating fast food, including insects in their diet (entomophagy), and trying foods produced with 3D printing. These areas were chosen because they capture both established eating habits (like fast food) and innovations that are still unusual or even surprising to most people (like insect-based or 3D-printed foods).

From the initial 1912 people who answered the questionnaire, only 608 declared that they were familiar enough with 3D food printing to give meaningful answers. As a result, this subgroup of 608 respondents became the focus for the analysis of attitudes toward 3D-printed food products (see Table 2).

This self-reported familiarity was used as a minimal screening criterion to avoid purely hypothetical responses, but it was not complemented by objective tests of knowledge or by more fine-grained measures of familiarity. As such, the study cannot fully control for differences in prior knowledge and awareness of 3D food printing across respondents, which may have influenced how they evaluated performance, effort and price.

As a theoretical background, the research was designed starting from a conceptual model well anchored in the theory regarding technology adoption and consumer behavior. The conceptual model proposed is based on seven working hypotheses (H1–H7) sustained by the literature review, in which we have proposed cause and effect relations between variables such as expected effort (EE)—expected performance (EP) and expected performance (EP), social influence (SI), facilitating conditions (FC), hedonic motivation (HM), perceived compatibility (PC)—perceived price (PP) and finally perceived price (PP) and behavioral intention (BI). For instance, we started from the idea that when people feel the effort involved is manageable, this shapes what they expect in terms of performance (H1), and those expectations naturally affect how they judge the price (H2). In the same way, factors like the opinions of others (H3), the resources and conditions available (H4), the enjoyment they obtain (H5), and how well the technology fits with their personal needs (H6) all play a role in how price is perceived. Ultimately, H7 suggested that people’s overall evaluation of price has a direct influence on whether or not they intend to embrace the technology.

### 4.2. Measurement Model Assessment

Before testing the structural relationships, the measurement model was evaluated to ensure internal consistency and validity. Reliability was assessed through Cronbach’s Alpha and Composite Reliability (CR), while convergent and discriminant validity were evaluated using Average Variance Extracted (AVE) and the Fornell–Larcker criterion.

When the model was tested using SEM analysis, however, the results showed it did not fit the data well enough. The main fit indicators (CFI = 0.808, TLI = 0.793, RMSEA = 0.095) all fell below the commonly accepted thresholds [63,64]. The χ^2^/df ratio was also too high (6.525 versus the recommended range of 3–5), and the significant *p*-value indicated notable differences between the estimated and observed covariance structures.

Looking more closely at the measurement model, two main sources of misfit emerged. First, the Hedonic Motivation construct performed poorly: the item loadings were low (0.38–0.46), AVE was below 0.50, and Composite Reliability fell short of the 0.70 benchmark. Unsurprisingly, the path HM → PPA was not significant (β = −0.137, *p* > 0.05), contradicting the original assumption. Second, Facilitating Conditions looked strong at the item level (loadings of 0.79–0.88) but had an almost negligible effect on PPA (β = 0.054, *p* > 0.05).

Because of this, keeping HM and FC would have only made the model more complicated without adding real explanatory value. Dropping them—along with item EE2, which showed weak loading—was both statistically and theoretically justified.

Importantly, the removal of HM and FC from the final SEM specification does not imply that these constructs are irrelevant for the adoption of 3D-printed food at a conceptual level. Rather, it reflects the fact that, in this empirical context, the specific items used to operationalize them did not achieve satisfactory psychometric performance or predictive power for price perception. Consequently, the final model should be viewed as a parsimonious, context-adjusted version of the original UTAUT2-based framework, retaining only those constructs that showed robust measurement properties and substantial explanatory value in the Romanian urban sample.

The revised model kept only the variables with solid empirical support (EE, EP, SI, PC, PPA, and BI). This streamlined version significantly improved the model fit, made the structure more coherent, and boosted its explanatory power and interpretability. In other words, the final model was not only simpler but also more reliable and statistically robust.

When analyzing the measurement model more closely, one issue stood out with the Expected Effort (EE) construct. Item EE2 (D5_2) showed a factor loading of only 0.394, much lower than the other items in the same construct (D5_1 = 0.594, D5_3 = 0.620, D5_4 = 0.774, D5_5 = 0.608). Since the recommended minimum for standardized loadings is 0.50 (Hair et al., 2019) [63], this indicated that EE2 contributed very little to the measurement of the latent variable and raised concerns about convergent validity.

If left in the model, EE2 would have undermined internal consistency and lowered both Composite Reliability (CR) and Average Variance Extracted (AVE). Removing it was therefore justified on statistical grounds and also helped improve the overall quality of the model, which in turn optimized the fit indices in the final version.

The results of the measurement model are summarized in Table 3, showing that the revised specification substantially improved the overall fit compared to the initial version.

Incremental indices such as CFI (0.931), TLI (0.923), NFI (0.909), and IFI (0.931) all exceeded the 0.90 threshold, which is generally accepted as indicating a good fit. The χ^2^/df ratio dropped to 3.868, which is comfortably within the acceptable range identified by Kline (2016) [64]. RMSEA also improved, coming in at 0.069—below the 0.08 limit, suggesting an acceptable fit. Parsimony indices (PNFI = 0.819 and PCFI = 0.839) were well above the minimum of 0.50, showing that the model was efficient relative to its complexity. Finally, the HOELTER index values confirmed adequate stability of the estimates, being above the 75 minimum, though still just below the optimal 200. Overall, these results point to a solid fit, supported by nearly all of the recommended indicators in methodological literature.

As part of the analysis, standardized factor loadings were also calculated to show how strongly each item is linked to the latent construct it was meant to measure.

Looking at Table 4, it is clear that all factor loadings exceeded the 0.50 minimum threshold [63], confirming that each item makes a meaningful contribution to its corresponding construct. Most loadings fell in the “good” range (>0.70), and some even reached the “excellent” level (>0.90). This was particularly the case for the Social Influence (SI) construct, which showed very high internal consistency. A handful of items—such as EE5, EP2, PPA4, and BI3–BI5—were in the “acceptable” range (0.60–0.69), but this did not pose any serious concerns.

In terms of reliability, Composite Reliability (CR) scores for all constructs were above the 0.70 benchmark, ranging from 0.815 for EE to 0.961 for SI, which confirms good to excellent internal consistency. Average Variance Extracted (AVE) values also met the minimum 0.50 criterion, ranging from 0.502 (BI) to 0.892 (SI). This demonstrates convergent validity, meaning that on average, more than half of the variance in the indicators is explained by the construct itself.

Cronbach’s Alpha values further confirmed the reliability of the measurement model, as all were above 0.70. SI (0.962) and EP (0.871) in particular stood out, showing excellent homogeneity among their items.

Taken together, Table 4 confirms that the measurement model meets the standard requirements for both reliability and convergent validity across all constructs. There were no items falling below the 0.50 threshold, and the smaller differences between “good” and “acceptable” indicators may be used in future refinements (for example, reevaluating EE5, EP2, PPA4, and BI3–BI5).

Discriminant validity was checked using the Fornell–Larcker criterion (Table 5). On the diagonal, the square roots of the AVE values for each construct are presented, while the off-diagonal elements represent Pearson correlations between constructs. According to Fornell and Larcker (1981), the diagonal value for each construct should be larger than any of its correlations with other constructs [68].

The results in Table 5 confirm this condition: each construct shared more variance with its own indicators than with those of any other construct. This indicates that the latent variables were sufficiently distinct from each other, both conceptually and empirically. In other words, each set of items measured what it was intended to measure, and not something else. At the same time, the correlation between Expected Effort and Expected Performance was relatively high (r = 0.716), which is conceptually plausible given that easy-to-use technologies are often perceived as more useful. To verify that this association did not create problematic multicollinearity or redundancy, we examined additional diagnostics: variance inflation factors (VIFs) were below 3.0 for all constructs, and the square roots of AVE values on the diagonal remained higher than any off-diagonal correlations. In UTAUT-type models, EE is often conceived as an antecedent of EP, which conceptually explains their strong positive association. These results suggest that EE and EP, while strongly related, still capture distinct aspects of the adoption process (perceived ease versus perceived benefits), justifying their simultaneous inclusion in the structural model. Although this correlation is relatively high, it remains below the conservative thresholds of 0.85–0.90 that are typically used to indicate problematic multicollinearity, so it is unlikely to undermine the validity of the estimates, although the EE–EP relationship should still be interpreted with an appropriate level of caution.

### 4.3. Structural Model Evaluation

After confirming the adequacy of the measurement model, the structural model was tested to evaluate the hypothesized relationships among constructs. Model fit indices indicated a good overall fit (χ^2^/df = 3.868; CFI = 0.931; TLI = 0.923; RMSEA = 0.069), consistent with recommended thresholds (Hair et al., 2019) [63].

Finally, the structural model was evaluated in AMOS. Table 6 shows that all hypothesized paths were statistically significant (*p* < 0.001). The regression coefficients (β) give a sense of the strength of these relationships. For instance, the effect of EE on EP was very strong (β = 1.518), while SI on PPA was the weakest (β = 0.239), though still significant. The Critical Ratio (C.R.) values for all paths were greater than ±1.96, confirming significance at the 95% confidence level [69].

The strongest path was between EE and EP (β = 1.518, *p* < 0.001), indicating that consumers’ perception of effort strongly shaped their perception of expected performance. The PPA → BI path also showed a substantial effect (β = 1.447, *p* < 0.001), meaning that a positive assessment of price–performance directly increased the intention to adopt 3D food printing. By contrast, SI → PPA (β = 0.239, *p* < 0.001) was the weakest link, suggesting that peer or social influence had a more modest role in shaping perceptions of price.

Overall, all hypotheses (H1–H7) were validated in the revised model, confirming the coherence of the theoretical framework and its empirical relevance. The optimized structure not only fit the data better but also provided stronger explanatory power and robustness for understanding consumer attitudes toward emerging food technologies.

### 4.4. Summary of Findings

The final model supports five of the seven originally hypothesized paths, confirming the mediating role of Price Perception (PP) and the predominance of utilitarian over hedonic and infrastructural factors. These results highlight that adoption of 3D-printed food among Romanian consumers is primarily driven by perceptions of performance, social influence, and compatibility, filtered through the lens of price fairness.

## 5. Discussion over the Results in Light of the Proposed Hypotheses

### 5.1. Overview of Key Findings and Theoretical Implications

The results of this study offer valuable insights into how functional, social, and contextual factors jointly shape consumer acceptance of 3D-printed food. Unlike traditional UTAUT2-based models, which generally emphasize direct effects of performance expectancy, effort, or social influence, the present analysis reveals that these drivers operate through a price-related evaluative mechanism. This demonstrates that the adoption of food innovations involves an additional layer of cognitive appraisal—one where consumers translate perceived benefits into behavioral intention by assessing price fairness and value-for-money considerations.

Consequently, price perception emerges not merely as an outcome of technological beliefs, but as a central interpretive filter in consumers’ decision-making process. This insight broadens the explanatory scope of the UTAUT2 framework and underscores the economic realism required when applying technology acceptance models to food-related contexts.

The following subsections discuss each hypothesis in detail, emphasizing how the observed relationships align with or diverge from previous empirical evidence and theoretical expectations.

### 5.2. Expected Effort and Performance (H1)

The confirmation of H1 shows that Expected Effort (EE) does indeed boost Expected Performance (EP). Simply put, if people feel that using 3D-printed food will be easy rather than complicated, they are more inclined to expect good results and real benefits from it. When the adoption process seems effortless, confidence in the technology’s usefulness naturally rises. This finding lines up well with what the main technology adoption theories, like the Technology Acceptance Model (TAM) and the Unified Theory of Acceptance and Use of Technology (UTAUT), have been saying for many years: people’s willingness to adopt new tech is shaped by a balance between how easy it feels to use and how much they believe it will actually help them.

Similar findings have been reported in the context of food innovations. For example, Lupton and Turner (2018) [70] showed that consumers were more open to 3D-printed food when the technology was perceived as user-friendly. Hartmann and Siegrist (2017) [71], also observed that reducing complexity in novel food technologies improved expectations of product performance.

Beyond 3D-printed food, similar mechanisms were identified in the adoption of cultured meat. Bryant and Barnett (2018) [72] found that when consumers understood the process as simple and accessible, their expectations of the product’s benefits increased. This parallel reinforces the idea that minimizing perceived effort acts as a driver of positive outcome expectations across different food innovations.

From a managerial perspective, this finding suggests that communication and marketing strategies for 3D-printed food should emphasize simplicity, convenience, and integration into everyday life. For example, highlighting how 3D printers can deliver ready-to-eat, customized meals with minimal input from the user could help increase both the perceived ease and the expected benefits.

Theoretically, the validation of H1 contributes to the literature by extending the TAM/UTAUT logic to the domain of food innovation adoption. It shows that Expected Effort is not only a barrier when perceived as high, but also a positive enhancer of outcome expectations when perceived as low. Thus, reducing perceived effort is essential not only for encouraging initial trial but also for shaping positive performance expectations, which are critical precursors of downstream constructs like price perception and behavioral intention.

### 5.3. Performance Expectancy and Price Perception (H2)

Validation of hypothesis H2 shows that performance expectations (PE) positively influence how consumers perceive price (PP) for 3D-printed food. In other words, when people believe that the technology provides clear results and real functionalities, they are more willing to consider the price as fair and justified. Basically, high performance expectations reduce the fear of paying too much and strengthen the balance between value and cost. This finding aligns with the existing research on food innovations. For instance, Hartmann and Siegrist (2017) [71] noted that consumers are more tolerant of premium pricing for innovative foods when they believe the product delivers tangible benefits such as improved nutrition or safety. Similarly, Lupton and Turner (2018) [70] found that strong performance expectations regarding personalization and convenience enhanced consumer willingness to pay more for 3D-printed meals.

Comparable mechanisms have been observed in related domains such as plant-based and cultured meat alternatives. Bryant and Barnett (2018) [72] reported that expectations of improved sustainability and ethical benefits influenced consumer perceptions of price fairness. Similarly, a systematic review by Onwezen et al. (2021) [73] synthesizes consumer acceptance across a wide spectrum of alternative proteins—including pulses, algae, insects, plant-based meat, and cultured meat—highlighting key drivers such as taste, health, familiarity, neophobia, and social norms.

From a practical perspective, this suggests that firms introducing 3D-printed food should link performance narratives to price justification. It means that satisfaction can also play such an important role in the 3D food intention to adopt the consumption of 3D-printed food [74]. Marketing communication could stress concrete performance advantages such as portion customization, enhanced nutritional accuracy, or efficiency in meal preparation. Positioning the technology as superior not only in novelty but also in delivering reliable benefits will help frame the price as rational and fair.

Theoretically, the confirmation of H2 strengthens the understanding that performance expectations operate as a key antecedent of price evaluation in food innovation adoption. This extends earlier findings from TAM/UTAUT research by demonstrating how performance expectancy does not only shape usage intentions but also moderates the financial evaluation of novel technologies. In the case of 3D-printed food, the perception of high performance creates a cognitive justification for higher prices, anchoring willingness to pay within perceived value frameworks.

### 5.4. Social Influence and Price Perception (H3)

Validation of hypothesis H3 shows that social influence (SI) has a positive impact on price perception (PP) in the case of 3D-printed food. Simply put, when people feel that people important to them—family, friends or social groups—support or approve the adoption of such products, they tend to consider the price as more reasonable. This means that price perception is not only an individual assessment, but is also shaped by social approval and group norms.

This result is in line with the role of subjective norms highlighted by adoption models, such as UTAUT, which emphasize that the opinions of reference groups strongly influence intentions and the way in which evaluations are formulated. In the context of novel food technologies, Lupton and Turner (2018) [70] showed that consumers’ willingness to accept and justify the price of 3D-printed meals increased when they believed such practices were socially endorsed. Similarly, Hartmann and Siegrist (2017) [71] found that peer influence mitigated skepticism toward novel food pricing.

Research on alternative proteins has also highlighted the importance of social influence. For instance, Onwezen et al. (2021) [73] reported that peer endorsement and social narratives shaped consumers’ perception of price fairness for insect-based and plant-based products. Similarly, Bryant and Barnett (2018) [72] emphasized that social acceptability was a critical factor in consumers’ evaluation of cost–benefit trade-offs.

From a managerial perspective, this result suggests that marketing strategies for 3D-printed food should be based on social proof, influencer support, and community stories. Highlighting positive testimonials, influencer endorsements, or collective benefits (e.g., shared sustainability contributions across social groups) can increase consumers’ willingness to accept price levels. At a theoretical level, the validation of H3 makes an additional contribution to the literature by showing that price perception is not only shaped by cognitive evaluations of product attributes, but also by social validation mechanisms. This highlights the importance of integrating social context into models of the adoption of disruptive food technologies. By recognizing that people rely on social cues to decide whether a price is justified, researchers can better understand the dynamics of the diffusion of 3D-printed food and other innovative technologies.

### 5.5. Facilitating Conditions and Price Perception (H4)

Rejection of hypothesis H4 shows that Facilitating Conditions (FC) did not have a significant impact on Price Perception (PP) for 3D-printed food. This means that any kind of facilitators like resources, infrastructure, or technical support does not have a real impact when consumers evaluate whether the price of these products is fair or acceptable. Instead, price judgments are more influenced by the perceived benefits of the product and in a certain extent by social dynamics.

This result distance itself from the conclusions formulated by the UTAUT model, where facilitating conditions are often associated with adoption behaviors. In food innovation research, some studies found partial relevance of contextual enablers, but not necessarily in relation to price evaluation. For example, Hartmann and Siegrist (2017) [71] emphasized that technological transparency and availability could improve general acceptance, but their study did not link these factors directly to perceptions of cost. Likewise, Lupton and Turner (2018) [70] reported that convenience and usability of 3D printers were relevant for adoption intentions, yet they did not translate into a more favorable perception of price.

Comparable outcomes are visible in studies on cultured meat and alternative proteins. Bryant and Barnett (2018) [72] found that while availability and infrastructure were preconditions for adoption, consumers’ judgments about price fairness were far more influenced by perceived performance, ethical considerations, and social endorsement. Similarly, Onwezen et al. (2021) [73] noted that although facilitating conditions (distribution, labeling, retail presence) supported awareness, they were insufficient to positively alter consumers’ perception of price.

From a managerial perspective, this result shows that firms cannot rely on the existence of infrastructure or logistics channels to justify higher prices for 3D-printed food. Even if consumer access and distribution are assured, price perception remains primarily linked to perceived value and social legitimacy, not to infrastructural availability. Consequently, marketers should focus on communicating concrete benefits and building social narratives, rather than highlighting enabling conditions.

From a theoretical perspective, the rejection of H4 highlights an important nuance: enabling conditions are not universally relevant to all dimensions of adoption. Although they may be essential for use and diffusion, they do not appear to directly influence financial valuations. This finding contributes to refining models of technology adoption by distinguishing between factors that determine intent to use and those that shape economic judgments such as price fairness.

Additionally, for many respondents 3D-printed food still represents a largely hypothetical option rather than a concrete purchase scenario. In such an early-stage context, infrastructural aspects are not yet salient when consumers evaluate price fairness: what matters more is whether the product itself appears beneficial and reasonably priced in principle. This helps explain why facilitating conditions did not exert a significant effect on price perception, even though they remain important for actual usage in more mature diffusion stages.

### 5.6. Hedonic Motivation and Price Perception (H5)

The statistical tests indicate that Hedonic Motivation (HM) does not have a significant positive effect on Price Perception (PP) in the context of 3D-printed food (path HM → PP not significant; HM items with weak loadings). In practical terms, experiential enjoyment, fun, or novelty do not translate into a more favorable judgment of price fairness at this stage of market diffusion. This null effect coheres with the measurement evidence (low standardized loadings and insufficient convergent validity for HM), suggesting that hedonic responses may be salient for sensory liking or initial curiosity, yet insufficient to justify price when consumers form value-for-money evaluations.

Findings in the 3D food printing literature often highlight hedonic and sensory angles, but primarily in relation to sensory acceptance or curiosity, not directly to price fairness. There are several reviews and empirical studies that emphasize novelty, sensory attributes, and experiential value in 3D-printed foods and adjacent domains, without consistently showing a direct pathway to price perception. Mantihal et al. (2019) [59], highlights the role of novelty/sensory in 3D printing, but does not establish a robust HM → PP relationship. In their study, Manstan et al. (2021) [75], confirm the role of sensory experience, meaning that a pleasant first contact (assessed by hedonic scales) improves the attitude towards 3D-printed food. However, there is no evidence that this positive consumption automatically translates into a more favorable price perception—the relationship remains indirect and unspecified. Similar findings were reported by Gârdan et al. (2021) [76], who showed that hedonic motivations play an essential role in shaping consumer experience with food delivery applications, although the translation into economic evaluations such as price fairness remains indirect.

Other authors [77] provide a critical review of 3D food printing, particularly in healthcare applications, and discuss benefits such as enhanced texture and personalized nutrition; while sensory and experiential features are noted, they are not positioned as direct drivers of price judgments. And finally, Brunner et al. (2018) [78], shows that affective elements can modulate attitude, but price perceptions are more closely related to utility/performance and norms. Taken together, these results suggest that, in the current early stage of 3D-printed food diffusion, hedonic reactions tend to shape curiosity, trial intentions and general attitude rather than economic evaluations such as price fairness. For most respondents, 3D-printed food still represents a largely hypothetical option, so their value-for-money judgments are anchored more in perceived performance and social legitimacy than in the “fun” or novelty of the experience. This provides a plausible explanation for the non-significant HM → PP path in our model.

From the perspective of adoption theories, UTAUT2 syntheses show that hedonic motivation generally has robust effects on attitudes/intention or intention to use, but not necessarily on economic evaluations such as PP (where expected performance and value justification matter more). In this respect, the paper of Alalwan et al. (2017) [79], shows that HM influences intention, but the translation to price fairness is contingent and often indirect. The systematic literature review made by Tamilmani et al. (2021) [31] shows that HM frequently appears as a predictor for intention/use, but not as a constant determinant of price perception.

From the point of view of the implications for the literature in the field, the non-significant result indicates that, in the adoption of 3D-printed food, economic evaluations (EEs) are primarily a function of performance expectations and social context (confirmed in H2/H3), while hedonic motivation may operate indirectly (e.g., through attitude or intention) or as a moderator (accentuating the effects of performance in contexts with high experiential salience). Thus, adoption models should more clearly decouple the hedonic role in price fairness from its role in attitude/intention, at least in the early stages of technological diffusion.

On the other hand, from the point of view of implications for practitioners/decision makers, it become obvious that campaigns that rely predominantly on the “wow-factor” and the novelty of the experience may increase curiosity and trial, but do not seem sufficient to justify the price. To improve PP, communication must explicitly support performance benefits (personalization, nutritional accuracy, efficiency, waste reduction) and social legitimacy (norms, social approval), using hedonic elements as support (design, point-of-sale experiences), not as an anchor of value. In the context of public policies, education and transparency interventions remain a priority to convert experiential interest into value perceptions.

As a final remark regarding the fact that HM → PP was not validated, while other determinants of PP were confirmed (e.g., EP → PP; SI → PP), supports the idea that price perception for 3D-printed food is formed mainly through utilitarian and social filters, and the hedonic dimension, although relevant for experience/attitude, does not provide a sufficient economic justification for price—at least at the current stage of consumer familiarity with the technology.

The statistical results showed that the hypotheses H4 (Facilitating Conditions → Price Perception) and H5 (Hedonic Motivation → Price Perception) were not confirmed in the tested model. None of these variables demonstrated significant explanatory power, and their retention would have unnecessarily complicated the model, without bringing any real interpretative value. Their elimination in the refinement stage thus contributed to improving the overall fit and parsimony of the model. The lack of support for Facilitating Conditions (H4) contrasts with a number of previous studies, which have emphasized the importance of infrastructure, resources, and technical support in shaping consumers’ perceptions of new technologies.

For example, Alalwan et al. (2017) [79] highlighted the importance of enabling conditions in the adoption of mobile banking services, and Venkatesh et al. (2012) [20] integrated them as a central element in the UTAUT2 model. A possible explanation for the difference found in the present case is that, in the context of 3D-printed food, enabling conditions are perceived by consumers as distant or irrelevant. They tend to pay more attention to perceived performance, price fairness and social acceptance, rather than to the technical or infrastructural factors that make the technology possible.

Similarly, the rejection of the Hedonic Motivation hypothesis (H5) is worth noting, as previous literature has often shown that pleasure, novelty and curiosity are important factors in the adoption of food innovations [59,71]. For example, Godoi et al. (2016) [80], suggested that consumers’ desire for exploration and playfulness can positively influence the acceptance of 3D-printed food in experimental contexts. However, the current results suggest that, in the urban market in Romania, hedonic aspects do not significantly influence price perception. This could indicate that although consumers recognize the novelty of 3D-printed food, price evaluation remains anchored in utilitarian factors such as quality, accessibility, and compatibility, rather than in elements of pleasure or amusement.

From a theoretical perspective, these results reinforce the idea that context plays a decisive role in the application of adoption models such as UTAUT2. Not all constructs have the same relevance in all technological domains, especially in the food sector, where utilitarian and social dimensions may weigh more than hedonic or infrastructural ones. For practitioners and policymakers, this implies that communication strategies should emphasize performance, compatibility, and social influence, and less on entertainment value or infrastructural ease, when seeking to strengthen consumer perceptions of 3D-printed food.

### 5.7. Perceived Compatibility and Price Perception (H6)

The findings provide strong support for H6, showing that Perceived Compatibility has a clear effect on how consumers judge the price of 3D-printed food. Put simply, when people feel that this kind of product “fits” their lifestyle, habits, and values, they are more likely to see the price as reasonable. In this sense, compatibility acts as a bridge that links technological innovation with consumer acceptance.

This aligns well with earlier work. Rogers’ Diffusion of Innovations theory, for instance, pointed out that technologies viewed as compatible with users’ everyday needs and experiences tend to spread faster [81].

More recently, empirical studies in food-related contexts have confirmed similar mechanisms: Candel (2001) [82] showed that meal convenience behaviors are heavily shaped by how compatible new solutions are with existing routines.

The positive validation of H6 also aligns with findings by Lupton and Turner (2018) [83], who demonstrated that consumer responses to digital food technologies depend strongly on the perceived congruence with personal lifestyles and cultural norms. In the same line, Godoi et al. (2016) [80] underlined that the success of 3D-printed food in consumer markets hinges on aligning product properties with existing expectations and usage patterns.

Taken together, these findings suggest that compatibility exerts a dual effect: it not only lowers perceived risk but also enhances the perceived fairness of price. In the context of 3D-printed food, this means that acceptance is facilitated when consumers feel the product can be easily integrated into daily consumption practices without requiring major behavioral adjustments.

From a managerial perspective, this implies that companies introducing 3D-printed food should prioritize positioning strategies that highlight compatibility—for example, by framing such products as extensions of familiar eating habits, or by linking them to culturally meaningful practices. Price acceptance is thus less a matter of absolute cost and more of how seamlessly the innovation fits with consumers’ existing mental models.

### 5.8. Price Perception and Behavioral Intention (H7)

The validation of H7 demonstrates that Price Perception (PP) exerts a positive influence on Behavioral Intention (BI) to adopt 3D-printed food. This finding emphasizes that consumers’ willingness to embrace such an innovation is directly conditioned by how fair and acceptable they perceive the price to be. When consumers view the price as justified relative to the value delivered, their likelihood of adoption increases substantially.

This result confirms a long-standing body of research that highlights the centrality of price fairness in shaping adoption decisions. Zeithaml (1988) [36] established that price is not only a monetary sacrifice but also a cognitive construct strongly linked to perceived value, showing that consumers’ judgments about whether a price is “fair” have enduring effects on behavioral outcomes. In the context of food innovations, Varela and Fiszman (2013) [37], demonstrated that perceived value is crucial in determining consumers’ willingness to try novel food products, with price perception operating as a decisive driver.

Specific to 3D-printed food, recent research reinforces this conclusion. For instance, Lupton and Turner (2018) [83] noted that skepticism toward 3D-printed food decreases significantly when consumers feel that the pricing corresponds to tangible benefits, such as personalization or nutritional control. Similarly, Godoi et al. (2016) [80], emphasized that while technological novelty initially drives curiosity, the long-term adoption of 3D-printed food is conditional on perceptions of economic rationality and price fairness.

Validating H7 has both theoretical and managerial implications. Theoretically, the results support the growing literature on value-based adoption models, where price perception plays a mediating role between perceived product features and adoption intention. At a managerial level, the message is clear: companies need to be very careful with pricing strategies so that consumers feel a balance between cost and the unique benefits offered. Communication should emphasize not only the novelty of 3D-printed food, but also its tangible value—whether we are talking about personalization, sustainability, or nutritional optimization.

### 5.9. Integrative Discussion and Theoretical Implications

Taken together, the results of the tested model offer a nuanced understanding of consumer adoption dynamics regarding 3D-printed food. The validation of H1–H3, H6, and H7 demonstrates that consumers’ adoption intentions are primarily shaped by a combination of expected performance, social influence, compatibility, and price perception. In contrast, the rejection of H4 (facilitating conditions) and H5 (hedonic motivation) suggests that traditional drivers such as infrastructure or pure enjoyment are not decisive in the specific case of 3D-printed food. This points to an adoption process that is more rational and value-based than affective or convenience-driven.

Theoretically, these findings align with and extend the Unified Theory of Acceptance and Use of Technology (UTAUT) framework (Venkatesh et al., 2003) [84] showing that not all constructs exert equal influence in the food technology domain. Similarly to the evidence provided by Godoi et al. (2016) [80], where consumer trust in 3D-printed food hinged on clear utility rather than on entertainment value, our findings indicate that price fairness and perceived usefulness are the pivotal anchors for acceptance.

The central role of price perception in mediating adoption confirms Zeithaml’s (1988) [36] classical insights on the dual nature of price as both sacrifice and value. More recent empirical studies further validate this mechanism: Lupton and Turner (2018) [83] demonstrated that consumer reluctance toward 3D-printed food decreases when price is perceived as commensurate with benefits such as personalization and nutritional optimization. In the same vein, Varela and Fiszman (2013) [37] emphasized that willingness to try innovative foods is contingent on a balance between novelty and perceived economic rationality.

This pattern is especially meaningful in the Romanian context, where food expenditures account for a relatively large share of household budgets and consumers have traditionally shown high price sensitivity for both staple products and innovations. In such settings, positive evaluations of performance or social endorsement do not automatically translate into adoption unless the perceived value-for-money is satisfactory. The strong mediating role of price perception therefore reflects not only a generic economic mechanism, but also the specific cultural-economic conditions of an emerging market in Eastern Europe, where income constraints, inflation episodes and trust in food quality are salient issues in everyday consumption decisions.

From a practical standpoint, the results underscore the importance of strategic communication and pricing policies for companies entering the 3D food printing market. Beyond showcasing technological novelty, firms must ensure that consumers perceive clear value propositions—whether linked to health benefits, customization, or sustainability. Involving consumers directly in the development process and clearly linking product benefits to cost has been shown to enhance acceptance of food innovations in the market [83].

In sum, this research contributes to the growing literature on technology adoption in the food sector by identifying price perception as the key determinant of behavioral intention toward 3D-printed food, while discounting the roles of hedonic enjoyment and infrastructural facilitation. It also provides actionable insights for industry players, who must focus on value communication and consumer trust-building strategies if they are to transform curiosity into long-term acceptance.

### 5.10. Managerial and Policy Implications

Overall, these findings offer valuable guidance for policymakers and industry stakeholders. Public institutions can use such insights to design educational and transparency campaigns that address skepticism toward food technologies, while firms should integrate consumer value communication into pricing and product development strategies. Future research could extend this framework by including trust, sustainability perception, or sensory quality as mediating variables, to refine the predictive capacity of technology adoption models in the food sector.

For manufacturers of 3D food printers, the results suggest the importance of designing equipment and ingredient systems that enable competitive portion costs and communicating clearly how operating expenses translate into end-user prices. Entry-level printer models, transparent pricing schemes (for both machines and cartridges) and communication that highlights long-term savings or efficiency gains can strengthen perceived fairness of price among professional users. Restaurants and catering businesses that consider introducing 3D-printed dishes can position them initially in premium or themed offerings where personalization, nutritional tailoring, visual uniqueness and reduced food waste are clearly visible to customers, so that any price premium is perceived as justified. In addition, restaurants can experiment with co-creation formats (e.g., allowing guests to choose shapes, decorations or nutritional profiles) that turn 3D printing into an experiential feature rather than a purely backstage technology. Food tech start-ups operating in this space may benefit from combining user-friendly interfaces and modular service packages with experiential marketing tools such as tasting events, pop-up installations or collaborations with chefs and influencers. These strategies can reduce uncertainty, build trust and allow consumers to directly experience the added value of 3D-printed foods before making purchase decisions.

## 6. Conclusions

### 6.1. Theoretical Implications

This study has examined consumer acceptance of 3D-printed food by testing a structural model grounded in the UTAUT framework [84]. Given its cross-sectional design and non-probabilistic, urban sample, the empirical model and findings should be interpreted as exploratory rather than definitive, providing context-specific insights that need to be corroborated in more representative national studies. The empirical findings confirm the significance of expected effort, expected performance, social influence, perceived compatibility, and price perception as determinants of adoption intentions. Conversely, facilitating conditions and hedonic motivation were not supported, suggesting that adoption of 3D-printed food is guided more by rational value assessments than by enjoyment or infrastructural convenience.

From a theoretical perspective, these results contribute to the literature on technology adoption in food innovation by refining the applicability of UTAUT constructs. In doing so, the study contributes explicitly to Eastern European food innovation research by showing how, in this regional context, functional expectations, social validation and compatibility judgments are filtered through price perception before translating into intentions to adopt 3D-printed foods. The results of the study extend prior observations by Godoi et al. (2016) [80] that consumer trust in 3D-printed food hinges on perceived utility rather than entertainment. Moreover, the central role of price perception aligns with Zeithaml’s (1988) conceptualization of price as both sacrifice and value (Zeithaml, 1988) [36], and supports empirical evidence from Lupton and Turner (2018) [83], who found that consumers were more willing to accept 3D-printed food when pricing reflected tangible benefits. Overall, the study offers robust exploratory evidence on how performance beliefs, effort expectations, social influence, compatibility and price perception shape behavioral intentions toward 3D-printed food in an emerging market, and its conclusions should be interpreted in light of the sampling and measurement limitations discussed above.

### 6.2. Managerial and Policy Implications in the Filed

The managerial implications are clear: companies entering the 3D-printed food market must prioritize transparent value communication and fair pricing. Essential to building trust and driving adoption intent is to clearly demonstrate how these products meet consumer needs—whether through personalization, nutritional optimization, or sustainability. As Guiné et al. (2020) [85] point out, the success of food innovation adoption depends largely on aligning product messages with consumer values.

### 6.3. Research Limitations and Future Directions

Although the present study offers novel insights into 3D-printed food acceptance in an Eastern European context, several limitations need to be acknowledged in terms of both internal and external validity. First, the data were collected using an online, non-probabilistic convenience sample, reaching predominantly younger, urban and digitally literate respondents. As a consequence, the findings cannot be generalized to the entire Romanian population, particularly to older age groups and rural residents with different socio-economic conditions and technology access. Future research should use probability-based or stratified sampling strategies, including offline modes of data collection, in order to obtain nationally representative samples and to compare urban versus rural patterns of acceptance.

Second, the study relied exclusively on self-reported data obtained from a cross-sectional questionnaire. Such measures are susceptible to common method bias and social desirability, especially when dealing with emerging technologies perceived as modern or environmentally friendly. While anonymity and careful item wording were used to mitigate these risks, future studies should consider complementary methods, such as behavioral experiments, choice-based conjoint tasks or longitudinal designs, which can capture actual trial and usage behavior and help bridge the well-known gap between intention and real behavior in the case of innovative foods.

Third, although respondents were screened for minimal familiarity with 3D food printing, familiarity was based on self-perception rather than on objective knowledge checks. Given that 3D-printed food remains an abstract and unfamiliar concept for most consumers, the absence of concrete stimuli (e.g., pictures, short descriptive videos, or examples of printed dishes) may have led some participants to answer based on vague associations rather than on a clear understanding of the technology. Future research should provide standardized stimuli and include explicit measures of prior knowledge and involvement, in order to disentangle the effects of informational clarity from those of underlying attitudes and beliefs.

Fourth, in terms of the conceptual model, two UTAUT2 constructs—facilitating conditions and hedonic motivation—had to be removed from the final SEM specification due to weak factor loadings, insufficient convergent validity and lack of explanatory power for price perception. This outcome points to limitations in the operationalization of these constructs in the specific context of 3D-printed food, rather than to their irrelevance in principle. It is possible that alternative item formulations, tailored more closely to food-related experiences (e.g., hedonic responses to concrete consumption episodes, or facilitating conditions referring to specific food service and kitchen infrastructures), would show stronger performance. Accordingly, future studies should re-examine facilitating conditions and hedonic motivation with refined scales and, where appropriate, test them as determinants of attitude or usage rather than as direct predictors of price perception.

Fifth, the present model intentionally focused on technological and socio-economic predictors (effort expectancy, performance expectancy, social influence, compatibility and price perception) and did not incorporate several psychological variables that are central in the literature on novel food acceptance. Factors such as food neophobia, perceived naturalness, safety and health concerns, environmental concern or trust in food system actors are known to shape both emotional responses and purchase intentions for innovative products. Integrating these variables—for example by combining UTAUT2 constructs with established scales such as the Food Neophobia Scale or perceived naturalness measures—would allow future models to capture more accurately the decision-making process highlighted in recent food choice research, including studies such as Abdullah et al. (2024) [58] on how food choices shape emotional experience and purchase intentions.

Finally, the dependent variable in this study was behavioral intention rather than observed adoption behavior. While intention represents a key proximal antecedent of behavior in many theoretical frameworks, the gap between intention and actual consumption can be substantial for emerging, still unavailable products like 3D-printed food. As 3D-printed food moves from experimental settings toward real market offerings, future research should incorporate behavioral indicators such as trial decisions, repeated use, willingness to pay and actual purchase data, and examine how the role of price perception evolves as consumers gain more experience with the technology.

Taken together, these limitations suggest several concrete avenues for further research: (i) extending sampling to more diverse populations and countries in Central and Eastern Europe; (ii) refining the operationalization of hedonic motivation and facilitating conditions for food-specific contexts; (iii) integrating food neophobia, perceived naturalness, safety and health concerns, environmental concern and trust into extended adoption models; and (iv) moving from intention-based to behavior-based designs, supported by realistic visual or sensory stimuli. Addressing these directions would strengthen both the internal and external validity of the findings and contribute to a more comprehensive understanding of 3D-printed food acceptance.

## Figures and Tables

**Table 1:** Sample structure.

Criterion		Respondents (Number)	%
Age of respondents	18–25 years	203	33.4
	26–35 years	172	28.3
	36–45 years	83	13.7
	46–55 years	93	15.3
	56–65 years	32	5.3
	Over 65 years	25	4.1
Last level of education completed	Middle School	14	2.4
	Secondary Education	255	41.9
	College	44	7.2
	Faculty	178	29.3
	Masters	103	16.9
	Doctorate	14	2.3
Personal income	Under RON 2200 *	127	20.9
	RON 2200–3500	105	17.3
	RON 3501–4500	120	19.7
	RON 4501–7000	142	23.4
	RON 7001–10,000	79	13.0
	Over RON 10,000	35	5.8
Residence	Urban	488	80.3
	Rural	120	19.7
Residence type	Apartment	406	66.8
	House	202	33.2
Gender of respondents	Male	300	49.3
	Female	308	50.7
Total		608	100.0

Note: * 1 RON ≈ 0.1978 EUR, thus 2200 RON ≈ 435.16 EUR [61].

**Table 2:** Measurement items and sources.

Construct/Item from Questionnaire	References
Expected effort—EE	Venkatesh, V., & Davis, F. D. (2000) [26]; Frank, M. P., & George, G. (2023) [62]
My interaction with the 3D-printed food could be clear and understandable.	
Interacting with the 3D-printed food does not require a lot of my mental effort.	
I find 3D-printed food could be easy to be consumed.	
I find it easy to use 3D-printed food as I please.	
Learning how to use 3D-printed food could be easy for me.	
Expected performance—EP	Venkatesh, V., Thong, J. Y., & Xu, X. (2012) [20]
Three-dimensional printed food could be useful in my daily life.	
Three-dimensional printed food could increase my chances of achieving things that are important to me.	
Using 3D-printed food could help me accomplish things more quickly.	
Using 3D-printed food could increase my productivity.	
Social influence—SI	Venkatesh, V., & Davis, F. D. (2000) [26]
People who are important to me could think that I should use 3D-printed food.	
People who are important in my life should use 3D-printed food.	
The people around me could be encouraging me to use 3D-printed food.	
Facilitating conditions—FC	Venkatesh, V., Thong, J. Y., & Xu, X. (2012) [20]
I could have the resources necessary to use 3D-printed food.	
I could have the knowledge necessary to use 3D-printed food.	
Three-dimensional printed food could be compatible with other foods/ways of cooking that I use.	
I could get help from others when I have difficulties using 3D-printed food.	
Hedonic motivation—HM	Palau-Saumell, et al., (2019) [21]
I believe that using 3D-printed food could be fun.	
I believe that using 3D-printed food could be enjoyable.	
I believe that using 3D-printed food could be very entertaining.	
It would be easy for me to feel better after I use 3D-printed food.	
Perceived compatibility—PC	Moore, G. C., & Benbasat, I. (1991) [57]; Yang et al. (2024) [27]
Using 3D-printed food could match my existing food preferences.	
Using 3D-printed food could align with my lifestyle and dietary needs.	
Three-dimensional printed food could be suitable for my living environment and daily routine.	
Price perception—PP	Palau-Saumell, et al., (2019) [21], Yang et al., 2024 [27]
The price of 3D-printed food could be an acceptable one.	
The price of 3D-printed food could be commensurate with the quality and benefits it provides.	
The price of 3D-printed food could be affordable for me.	
The price of 3D-printed food could be competitive compared to other food alternatives on the market.	
Behavioral intention toward 3D food usage—BI	Venkatesh, V., & Davis, F. D. (2000) [26], Venkatesh, V., Thong, J. Y., & Xu, X. (2012) [20]
Assuming I will have access to the 3D-printed food, I intend to use it.	
Given that I could have access to the 3D-printed food, I predict that I would use it.	
I intend using 3D-printed food in the future.	
I will always try to use 3D-printed food in my daily life.	
I plan to use 3D-printed food frequently.	

**Table 3:** Model Fit Indices and Minimum Acceptable Thresholds.

Indicator	Obtained Value	Minimum Acceptable Threshold (Literature)	Source	Interpretation
χ^2^/df (CMIN/DF)	3.868	≤5 (acceptable)	Kline (2016) [64]	Acceptable
CFI (Comparative Fit Index)	0.931	≥0.90 (acceptable)	Hair et al. (2019) [63]	Good
TLI (Tucker–Lewis Index)	0.923	≥0.90 (acceptable)	Hair et al. (2019) [63]	Good
NFI (Normed Fit Index)	0.909	≥0.90	Hu & Bentler (1999) [65]	Good
IFI (Incremental Fit Index)	0.931	≥0.90	Hair et al. (2019) [63]	Good
RMSEA (Root Mean Square Error of Approximation)	0.069	≤0.08 (acceptable)	Hu & Bentler (1999) [65]	Acceptable
PNFI (Parsimony NFI)	0.819	≥0.50	Mulaik et al. (1989) [66]	Very good
PCFI (Parsimony CFI)	0.839	≥0.50	Mulaik et al. (1989) [66]	Very good
HOELTER (0.05)	182	≥75 (acceptable); ≥200 (good)	Hoelter (1983) [67]	Almost good
HOELTER (0.01)	194	≥75 (acceptable)	Hoelter (1983) [67]	Good

Sources: Hair et al. (2019) [63]; Kline (2016) [64]; Hu & Bentler (1999) [65]; Mulaik et al. (1989) [66]; Hoelter (1983) [67].

**Table 4:** Standardized Factor Loadings for the Measurement Model.

Construct	Item	Standardized Loading *	CR **	AVE ***	Cronbach’s Alpha
EE	EE1 (D5_1)	0.792	0.815	0.527	0.770
	EE3 (D5_3)	0.720			
	EE4 (D5_4)	0.771			
	EE5 (D5_5)	0.607			
EP	EP1 (D6_1)	0.815	0.859	0.605	0.871
	EP2 (D6_2)	0.679			
	EP3 (D6_3)	0.844			
	EP4 (D6_4)	0.763			
SI	SI1 (D7_1)	0.947	0.961	0.892	0.962
	SI2 (D7_2)	0.948			
	SI3 (D7_3)	0.939			
PC	PC1 (D10_1)	0.752	0.850	0.655	0.859
	PC2 (D10_2)	0.841			
	PC3 (D10_3)	0.832			
PP	PP1 (D12_1)	0.785	0.825	0.542	0.735
	PP2 (D12_2)	0.752			
	PP3 (D12_3)	0.761			
	PP4 (D12_4)	0.639			
BI	BI1 (D13_1)	0.768	0.833	0.502	0.902
	BI2 (D13_2)	0.779			
	BI3 (D13_3)	0.685			
	BI4 (D13_4)	0.628			
	BI5 (D13_5)	0.670			

Note: * Minimum threshold for standardized loadings ≥ 0.50; ** Minimum threshold for CR ≥ 0.70; *** Minimum threshold for AVE ≥ 0.50. Source: Hair et al. (2019) [63].

**Table 5:** Fornell–Larcker Criterion Matrix for Discriminant Validity.

Construct	EE	EP	SI	PC	PP	BI
EE	0.726					
EP	0.716	0.778				
SI	0.268	0.255	0.945			
PC	0.575	0.558	0.188	0.809		
PPA	0.715	0.726	0.565	0.728	0.736	
BI	0.705	0.658	0.537	0.676	0.677	0.709

**Table 6:** Standardized Regression Coefficients (β) and *p*-values for Structural Model Hypotheses (AMOS results).

Hypothesis	Tested Relationship	β (Estimate)	*p*-Value	Critical Ratio (C.R.)	Result
H1	EE → EP	1.518	<0.001	15.265	Supported
H2	EP → PP	0.353	<0.001	12.900	Supported
H3	SI → PP	0.239	<0.001	12.707	Supported
H6	PC → PP	0.373	<0.001	12.517	Supported
H7	PP → BI	1.447	<0.001	13.559	Supported

## Data Availability

The original contributions presented in this study are included in the article. Further inquiries can be directed to the corresponding authors.

## Abbreviations

The following abbreviations are used in this manuscript:

3D	Three dimensions
UTAUT2	Unified Theory of Acceptance and Use of Technology, second version
NASA	National Aeronautics and Space Administration
MIT	Massachusetts Institute of Technology
4D	Four dimensions
5D	Five dimensions
TAM	Technology Acceptance Model
EE	Expected Effort
EP	Expected performance
PP	Price perception
SI	Social Influence
PC	Perceived Compatibility
BI	Behavioral Intention
TRA	Theory of Reasoned Action
TPB	Theory of Planned Behavior
TTF	Task–Technology Fit
SCT	Social Cognitive Theory
TOE	Technology–Organization–Environment Framework
IDT	Innovation Diffusion Theory
MM	Motivational Motivation
MPCU	Model of PC Utilization
FC	Facilitating conditions
HM	Hedonic motivation
SEM	Structural Equation Modeling
CFI	Comparative Fit Index
TLI	Tucker–Lewis Index
AVE	Average Variance Extracted
CR	Composite Reliability
NFI	Normed Fit Index
RMSEA	Root Mean Square Error of Approximation
IFI	Incremental Fit Index
PNFI	Parsimony NFI
PCFI	Parsimony CFI
AMOS	Analysis of Moment Structures
